# Effects of repetitive transcranial magnetic stimulation on cognitive function and hormone levels in early stroke patients with low thyroid hormone levels

**DOI:** 10.3389/fnagi.2024.1460241

**Published:** 2024-10-16

**Authors:** Hong Li, Jiang Ma, Ziqiang Song, Xiaolin Tao, Yan Xing, Feng Zhang

**Affiliations:** ^1^Department of Rehabilitation Medicine, Shijiazhuang People’s Hospital, Shijiazhuang, China; ^2^Physical Education College, Hebei Normal University, Shijiazhuang, China; ^3^Department of Rehabilitation Nursing, Shijiazhuang People’s Hospital, Shijiazhuang, China; ^4^Department of Rehabilitation Medicine, The Third Hospital of Hebei Medical University, Shijiazhuang, China

**Keywords:** stroke, repetitive transcranial magnetic stimulation, thyroid hormone, cognitive function, HPT axis, rehabilitation

## Abstract

**Background:**

This study aimed to observe the effects of repetitive transcranial magnetic stimulation (rTMS) on cognitive function and thyroid hormone levels in early older stroke patients with low thyroid hormone levels, and to investigate the correlation between the changes in thyroid hormone levels and the improvements in cognitive function after stroke.

**Methods:**

Forty older stroke patients who met the inclusion criteria were recruited and randomized into a magnetic-stimulation group (rTMS group) and a sham-stimulation group (Sham group). The rTMS group received low-frequency true stimulation and the Sham group received low-frequency sham stimulation. Patients’ cognitive scores, activity of daily living(ADL) scores, and their levels of triiodothyronine (T3), free triiodothyronine (FT3), thyroxin (T4), free thyroxine (FT4), and thyroid stimulating hormone (TSH) were assessed before the intervention, after the 4-week intervention, and after an additional 4 weeks of follow-up; Repeated measurement analysis of variance was used to compare the changes of each index in the two groups at different times and the correlations between patiens’ cognitive function scores and their changing hormone levels were subsequently investigated.

**Results:**

Thirty-one patients were included in this study: 16 patients in rTMS group and 15 patients in the Sham group. Repeated-measures ANOVA showed that patients’ T3,FT3 and TSH levels tended to increase at 4-week intervention and at the follow up (*p* < 0.05), and that the rTMS group had a better effect on improving T3 than the Sham group (F_group_ = 5.319, *p* = 0.028); The cognitive scale at different time points in both groups showed an upward trend (*p* < 0.05), and the MoCA, DSF, DSB scores in the rTMS group were statistically higher than those in the Sham group at the end of the 4-week intervention and at the follow-up (*p* < 0.05); The changes in the levels of T3 before and after 4-week intervention were positively correlated with the changes in the MoCA scores (*r* = 0.638, *p* < 0.05). And the difference in T3 level change was positively correlated with the difference in delayed recall, attention and naming score change (*r* = 0.562, *p* < 0.05; *r* = 0.562, *p* < 0.05; *r* = 0.531, *p* < 0.05); and the difference in FT3 level change was positively correlated with the visuospatial and executive function (*r* = 0.514, *p* < 0.05).

**Conclusion:**

Repetitive transcranial magnetic stimulation improved cognitive function and elevated T3 levels in older patients with post-stroke cognitive dysfunction who had low thyroid hormone levels. Within the normal range, increases in T3 levels are positively correlated with changes in cognitive function.

## 1 Introduction

Stroke is characteristic by high morbidity, mortality and disability, and is the second leading cause of death and the third leading cause of disability worldwide (Dee et al., 2020; Kefale, 2019). Post-stroke cognitive impairment (PSCI), as one of the most important dysfunctions, has an incidence rate of up to 80% (Sun et al., 2014). PSCI refers to a series of syndromes characterized by cognitive dysfunction that meet the diagnostic criteria of cognitive dysfunction through clinical symptoms and functional evaluation, especially various types of cognitive impairment that occur after stroke (Huang et al., 2022; Wang et al., 2021). PSCI is closely related to the poor functional prognosis of stroke patients, and seriously affects individual’s ability to self-care and achieve re-occupation. The probability of dementia in stroke patients with cognitive impairment will increase by 4–12 times if it is not detected and treated quickly. Post-stroke dementia, greatly increases the difficulty of patient rehabilitation and seriously reduces the quality of life of patients in addition to imposing a heavy burden on individuals, families, and the society (Renjen et al., 2015; Yang et al., 2024). Therefore, early prevention and targeted mitigation of risk factors that lead to or exacerbate cognitive impairment are particularly critical.

The mechanism of PSCI occurrence is complex and has multiple influencing factors. Although it is known that damage to neuroanatomical structures related to cognitive function that is caused by stroke will directly lead to cognitive dysfunction, recent progress in brain research and the neuroendocrine mechanisms on the functional prognosis of stroke. It has been demonstrated that, as a stress event, stroke may activate the neuroprotective mechanism of the body. It can reduce the oxygen consumption and metabolic rate of the body through self-regulation of endocrine function, such as reducing the level of thyroid hormone, especially T3 level, which is conducive to the repair of the injured site. With the recovery of the disease, the serum thyroid hormone level will increase, but there will be differences between different individuals. Compared with the young people, the decrease of the physiological thyroid hormone level in the elderly after stroke may last longer resulting in dysfunction of the hypothalamic–pituitary-thyroid axis (HPT axis) which further aggravates cognitive impairment (Gkantzios et al., 2023; Lei et al., 2019; Murolo et al., 2022). Furthermore, older patients with post-stroke cognitive dysfunction who also have low thyroid hormone levels have been shown to have especially poor functional levels and worse prognoses (Li et al., 2021; Mei et al., 2022). Because there are no standardized clinical guidelines for the use of medications in patients who have abnormal thyroid hormone levels due to non-thyroidal diseases but not meeting the diagnostic criteria for hypothyroidism, it is unclear whether non-pharmacological interventions are indicated in these patients. Given the adverse effects of persistent low thyroid hormone levels in patients with post-stroke cognitive dysfunction (Gkantzios et al., 2023; Lei et al., 2019; Li et al., 2021; Mei et al., 2022; Murolo et al., 2022), here is a clinical need to identify a safe and effective way to intervene early in such patients to improve their functional prognosis and quality of survival.

As a non-invasive and safe treatment, rTMS has been increasingly recognized and accepted. It has been clinically used in the treatment of a variety of neurological and mental diseases. Existing studies have demonstrated that rTMS stimulation of Dorsal Lateral Prefrontal Cortex (DLPFC) can improve cognitive dysfunction after stroke by promoting synaptic plasticity, improving cerebral blood flow and cerebral metabolism, increasing cortical and subcortical functional connections and other neural mechanisms (Gao et al., 2023; Gong et al., 2023; Wang and Voss, 2015; Yang et al., 2015). Some studies have also found that rTMS can elevate thyroid hormone levels in healthy older adults as well as in stroke patients (Ma et al., 2021; Mei et al., 2022; Ren et al., 2017). We therefore questioned whether rTMS could similarly improve cognitive function in patients with PSCI who have low thyroid hormone levels, in addition to promoting the recovery of thyroid hormone levels. In conclusion, we aimed to observe the effects of rTMS on cognitive function and thyroid hormone levels in older patients with post-stroke cognitive dysfunction accompanied who have low thyroid hormone levels, and to provide a safe and effective therapeutic option for the clinical rehabilitation of this population.

## 2 Materials and methods

### 2.1 Patient characteristics

The patients in this study were older stroke patients who treated in the Department of Rehabilitation Medicine of Shijiazhuang People’s Hospital between October 2019 and December 2020. The inclusion criteria required that patients met the following conditions: (1) they provided signed informed consent and acceptance of cooperation; (2) Based on the diagnostic criteria revised in the 4th National Conference on Cerebrovascular Disease in 1995, and at the same time, confirmed by CT or MRI for the Stroke patients with first-onset, unilateral hemispheric lesions; (3) they showed stroke recovery (14 days ≤6 months) and stable vital signs; (4) they were at least 60 years of age; (5) they were right-handed; (6) they obtained a Montreal Cognitive Assessment (MoCA) score < 26; (7) Passed the safety screening for rTMS, and met the safety criteria for participating in the rTMS interventions; and (8) their serum thyroid hormone levels were lower than the normal reference value, but did not meet the diagnostic criteria for thyroid disease (Mei et al., 2022). The exclusion criteria were as follows: (1) previous thyroid disease; (2) dementia; (3) severe functional impairment of important organs; (4) severe mental and psychological disorders; (5) tetraplegia; (6) aphasia, hearing and comprehension disorders; (7) the presence of metal foreign bodies; (8) claustrophobia; (9) previous epilepsy; (10) severe sleep disorders; or (11) progressive exacerbation of the disease. Patients dropped out of the study if (1) they were patients who were uncooperative in completing the rTMS or sham stimulation treatment; (2) they were uncooperative in completing the cognitive assessment or (3) they withdrew from the treatment due to other reasons.

**Figure 1:**
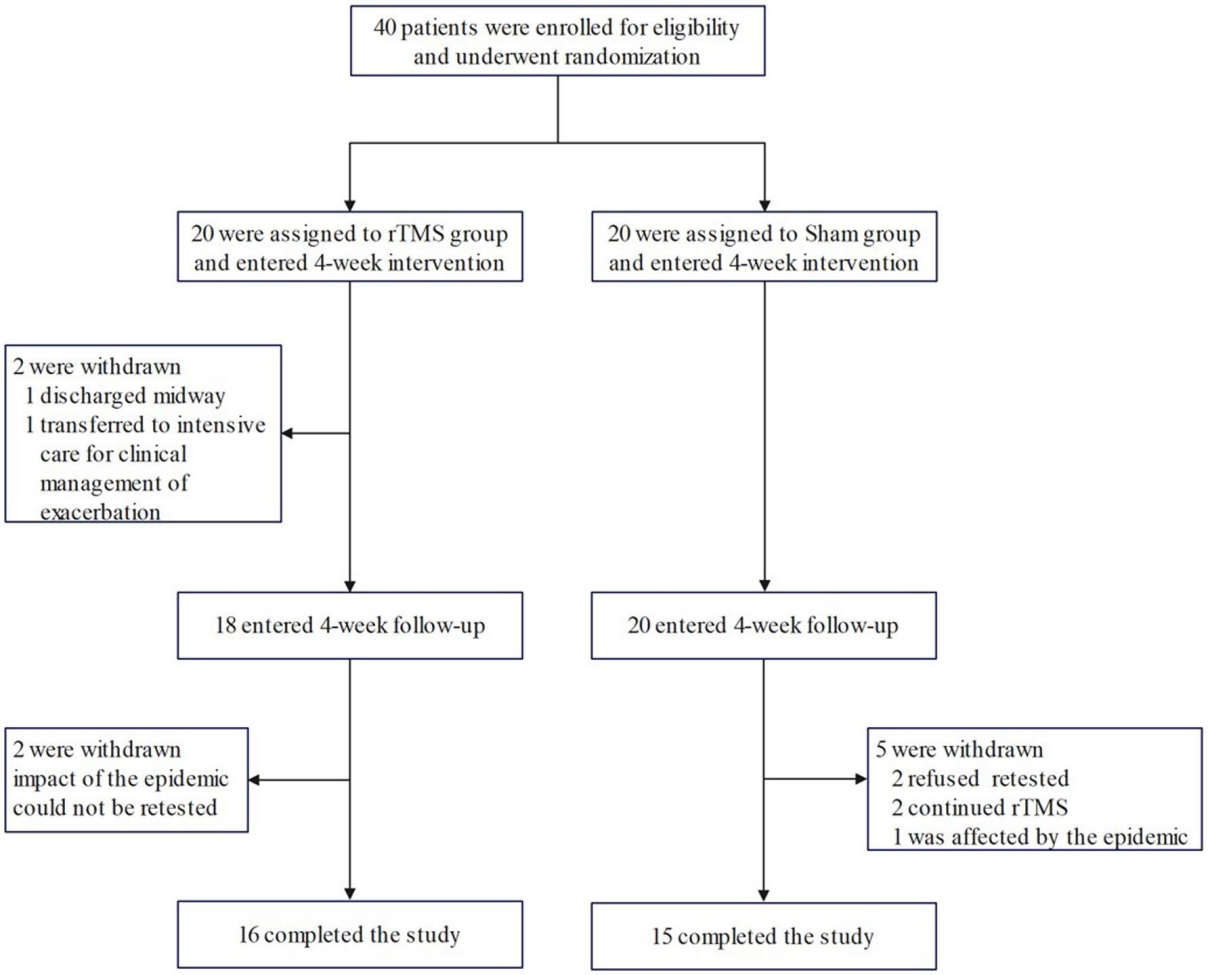
The flowchart of patient recruitment.

According to the random number table method, 40 patients were randomly divided into rTMS group (*n* = 20) and Sham group (using the same parameters as the rTMS group, but differently placed to not play a therapeutic role; *n* = 20) according to the order of consultation. Valid and complete data were obtained from 31 of these patients (16 patients in the rTMS group, and 15 patients in the Sham group) as 9 patients did not complete the current study due to the epidemic; among these 9 patients, 4 patients were in the rTMS group (1 patient was discharged halfway through the study, 1 patient was transferred to intensive care for clinical treatment for aggravation (not related to this study), and 2 patients could not be retested due to the impact of the epidemic), and 5 patients in the Sham group (2 patients refused to have their blood drawn for testing at the time of follow-up, 2 patients continued to undergo treatment in other hospitals for rTMS and tDCS, respectively, during the follow-up period, and 1 patient could not be retested due to the impact of the epidemic). The patient recruitment flowchart is shown in Figure 1.

### 2.2 Procedure

The information that was collected from all patients before the intervention included their age, gender, years of education, stroke type, disease duration, past medical history, and handedness. All enrolled patients were strictly screened with regard to the safety of repeated transcranial magnetic stimulation, and the indications and contraindications of transcranial magnetism were strictly controlled. An rTMS stimulator (manufacturer: Shenzhen Yingzhi) was then used to measure and determine each patient’s motor threshold (MT) and a magnetic stimulation programme was formulated. The patients in both the rTMS and Sham groups received the same basic treatments, including conventional medication, conventional rehabilitation, and cognitive function; rTMS treatment was added to the treatments given to the rTMS group, and sham stimulation was added to the treatments given to the Sham group. Cognitive function scores, activities of daily living (ADL) scores, and thyroid hormone indexes were evaluated before the intervention, at 4 weeks (immediately after the intervention concluded), and at follow-up (4 weeks after the intervention concluded).

### 2.3 Conventional rehabilitation

#### 2.3.1 Pharmacological treatment

Patients received appropriate pharmacological treatment for their underlying diseases, such as medications to manage their nerve nutrition, blood pressure, blood sugar, and blood lipids, in addition to drugs to prevent secondary injury.

#### 2.3.2 Conventional rehabilitation therapy

Patients received therapy that used nerve facilitation technology and motor re-learning to carry out conventional rehabilitation training for the hemiplegic side limb and to help them with turning over, sitting up, standing up, weight-bearing of the affected limb, walking training, balance training, and occupational therapy (Ni et al., 2018; Pepping, 2014; Yan et al., 2018) Patients undertook one 40-min session of training per day, 5 days per week, for 4 weeks in total.

### 2.4 Cognitive training protocol

Routine Cognitive Function Training (Ni et al., 2018; Pepping, 2014) was provided according to patient’s cognitive function at the time of consultation, targeted training is provided, which mainly included the following: (1) attention training—using visual tracking and linking games; (2) memory training using photos, pictures and auditory stimuli to repeatedly practice the processes of recognition, retelling, recall and re-recognition; (3) orientation training involved setting up a particular environmental scene and eliciting responses about time, place, people, and the location of related objects; (4) visual and spatial training - using jigsaw puzzles and object recognition; (5) executive training that involved setting up specific tasks (e. g., manual activities) and practicing independent thinking by planning, adjusting and completing them independently; (6) judgement and reasoning skills training that involved using puzzles and games and combining the background to complete the blank scenes to practice their logical reasoning ability. Patients undertook one 30-min session of training per day, 5 days per week, for 4 weeks in total.

### 2.5 rTMS protocol

#### 2.5.1 Safety screening

Before the intervention, each patient was screened with regard to rTMS safety during which his or her complete clinical history, past history and epilepsy history of the patient were fully determined; the indications and contraindications for rTMS were strictly controlled. rTMS contraindications included the presence of metal objects or devices in the skull or on the scalp, cardiac pacemakers, medical implantable devices, a history of epilepsy, pregnancy, implants in the body, or claustrophobia. Strictly in accordance with the instructions for the use of transcranial magnetism for standardized operation, there was detailed communication with the patients before the intervention to ensure that they had an objective understanding and awareness of the procedure, and each patient was instructed not to move his or her head during the treatment process, so as to avoid positional shifts affecting the therapeutic efficacy (Fox et al., 2012; Lefaucheur et al., 2014).

#### 2.5.2 Determination of motor threshold (MT)

Before the intervention began, the MT was measured and determined for each patient by applying the electrode sheet to the patient’s right adductor muscle, connecting the electrode sheet to the multichannel physiological signal recorder to collect EMG signals, and then stimulating the primary motor area of the left side of the brain with a single pulse of TMS (Schecklmann et al., 2020). The motor evoked potentials that were recorded on the EMG acquisition software and the contraction of the right adductor muscle were observed, and at least five out of ten consecutive stimulations had an amplitude greater than the minimum stimulation intensity of 50 μV, which can be regarded as the motor threshold MT (Fisicaro et al., 2019; Fox et al., 2012; Lefaucheur et al., 2014).

#### 2.5.3 Implementation of rTMS and sham interventions

Previous studies have shown that DLPFC is an effective target of rTMS for the treatment of post stroke cognitive impairment, and stimulation of this region can improve the cognitive function of stroke patients (Balconi, 2013; Fisicaro et al., 2019; Wang et al., 2024). In addition, other studies have found that stimulating the DLPFC region of older people with cognitive decline using rTMS can simultaneously improve cognitive function and thyroid hormone levels (Ma et al., 2021; Mei et al., 2022; Ren et al., 2017). So we chose DLPFC for our study. A transcranial magnetic stimulator and a standard figure-of-eight coolable coil were used, and the brain stimulation area localization method was based on the International 10–20 system of EEG and located at the Dorsal Lateral Prefrontal Cortex (DLPFC) locus (Balconi, 2013; Fisicaro et al., 2019). All parameters of the magnetic stimulation programme were set regardless of a patient’s treatment group (stimulation frequency 1 Hz, stimulation intensity 90% MT, stimulation duration: 20s, interval per cycle: 3 s; this treatment was performed for 20 min once per day, 5 times per week, over 4 weeks of continuous treatment). The placement of the coil differed between the two groups: the rTMS group was treated with the coil placed across the target area of patient’s healthy DLPFC, whereas the Sham group was treated with the coil placed perpendicular to the scalp to give a pseudo stimulation but had no therapeutic effect (Lefaucheur et al., 2014). The patients were told to remain relaxed, to avoid moving, and to ask and observe the patients promptly.

### 2.6 Outcome measures

Patients’ cognitive function scores, ADL scores, and thyroid hormone levels were assessed 1 day before the intervention, 4 weeks after the intervention began, and at follow-up assessment conducted 4 weeks after the treatment intervention ended.

#### 2.6.1 Cognition assessment

This assessment included the MoCA test, the verbal fluency test (VFT), and the digital span test.

##### 2.6.1.1 The montreal cognitive assessment (MoCA)

The MoCA scale is one of the most commonly used scales for assessing cognitive impairment in clinical practice. It assesses various cognitive domains-including alternating connectivity test (1-A-2-B-), orientation, visuospatial and executive functions (cube, clock), memory, attention, abstract thinking, language, naming, etc. The MoCA scale has a total score of 30, with cognitive impairment defined as a score below 26 points. When the patient’s number of years of education is less than or equal to 12 years, 1 point will be added to the test results. The total time required for this assessment does not exceed 10 min (Nie et al., 2012).

##### 2.6.1.2 The verbal fluency test (VFT)

Verbal sphere fluency was assessed using the VFT test (Henderson et al., 2023). This test requires the patient to name as many foods as possible within 1 min, and is scored according to the number of correct responses given by the subject. The instruction was given, “Now please list the names of as many foods as you can in 1 min, “was given, and a stopwatch was used to time the test, with the timing beginning after the initial instruction was given and the patient confirmed that he or she understood the instructions. The names of the foods listed by the subject were recorded sequentially; if the subject paused for more than 15 s, the instructions were repeated and then the patient was instructed to continue the list, and the time was counted. If patients stopped listing items before 1 min they were encouraged to try to continue.

##### 2.6.1.3 Digital span testing

Digital span testing gives digital span forward (DSF) and digital span backward (DSB) (Gao et al., 2023). Starting with a short string of digits with fewer digits, the tester first reads out a string of digits at a constant speed, and then asks the patient to repeat the digits in the order in which they were read. The tester gradually increases the number of digits until the patient cannot recite the string correctly, and the patient’s score is the highest number of digits that they correctly recited. DSB scores are obtained in an equivalent way but with the patients reciting the digits in the reverse order to which they were read by the tester.

#### 2.6.2 ADL scores

The Modified Barthel index (MBI) scale was used to assess the patient’s ability to perform activities of daily living (Pournajaf et al., 2023), and this was evaluated primarily on the basis of the patient’s actual performance, not on the basis of what the patient might be able to do or on the results of questioning. The standard evaluation includes ten (or eleven) basic components such as eating, dressing, and walking (or using a wheelchair), and the basic rating scale allows each activity to be rated on a 5-point scale, with different levels representing different degrees of self-dependence and self-care ability, ranging from the lowest degree at level 1 to the highest degree at level 5. A total score of 100 is considered normal, a total score of ≥60 is considered as basic self-care, between 41 and 59 is considered as partially dependent, a total score 21–40 is considered as mostly dependent, and a score of 20 or below is considered as totally dependent.

#### 2.6.3 Serum levels of thyroid hormones and thyroid stimulating hormone (TSH)

All patients had their blood collected, after fasting, on three occasions: in the morning of the day before the intervention began, 4 weeks after the intervention began, and at an 4-week follow-up visit conducted 4 weeks after the intervention concluded. Approximately 10 mL of blood was collected from the vein and injected into a procoagulant vacuum tube, which was left at room temperature for 1 h. The serum was separated by centrifugation at 3000 r/min for 10 min. During the assessment, we first checked that the relevant instruments were in good working condition, and then carried out the tests in strict accordance with the reagents’ instructions. The following hormones were measured using electrochemiluminescence: T3, T4, FT3, FT4, and TSH (Ma et al., 2021; Ren et al., 2017).

### 2.7 Data analysis

SPSS statistical software, version 25.0 was used to perform statistical analysis. P–P Plots was used to test the normality of all continuous variables. Normally distributed variables were presented as mean ± standard and non-normally-distributed variables were presented as median (25^th^ percentile, 75^th^ percentile). Age, disease course, and years of education were compared between the two groups using two-sample independent t-test, whereas gender, lesion side, and types of stroke were compared using the Chi-squared test. Using repeated measures ANOVA to analyze the changes in various indicators between two groups at different time points and the differences between groups. Mann Whitney U test was used to compare the changes of cognitive function and thyroid hormone levels (after 4 weeks of intervention pre intervention) between the two groups. Spearman correlation analysis was performed to assess the correlation between the post-treatment change in thyroid hormone levels and cognitive function scores (posttest–pretest) in the rTMS group, *p* values<0.05 were considered indicative of statistical significance.

## 3 Results

### 3.1 Comparison of general data between the two groups

Data from 31 patients (16 in the rTMS group and 15 in the Sham group) were analysed in this study. Before the intervention, there were no significant differences between the rTMS and Sham groups with respect to gender, age, disease course, side of lesion, types of stroke, or years of education (*p* > 0.05) (Table 1).

**Table 1:** Comparative analysis of the results of the general data of the two groups of patients.

Group	N	Gender		Age	Disease course	Lesion side		Types of stroke		Years of education
		Male	Female			Left	Right	Cerebral infarction	Cerebral haemorrhage	
rTMS	16	9	7	65.38 ± 3.26	26.94 ± 9.71	6	10	11	5	9.88 ± 3.01
Sham	15	8	7	66.13 ± 4.10	24.87 ± 8.58	7	8	10	5	10.13 ± 2.75
*P*		1		0.572	0.535	0.722		1		0.805

rTMS, repetitive transcranial magnetic stimulation.

### 3.2 Repeated measurement analysis of variance results of cognitive function between the two groups before and after intervention and at follow-up

Repeated measurement analysis of variance showed that MoCA, DSF and DSB scores had interaction between group and time (F_time*group_ = 7.631, *p* = 0.002; F_time*group_ = 5.479, *p* = 0.01; F_time*group_ = 4.114, *p* = 0.027). Further separate effect analysis showed that there were significant differences in MoCA, DSF and DSB scores between the two groups at different time points, and MoCA (*P*_post_ = 0.044; *P*_follow-up_ = 0.038), DSF (*P*_post_ = 0.027; *P*_follow-up_ = 0.048), DSB (*P*_post_ = 0.033; *P*_follow-up_ = 0.045) in rTMS group at the time of intervention and follow-up, which were better than those in sham group. The scores of MoCA, DSF and DSB in rTMS group after intervention and follow-up were significantly different from those before intervention (*p* < 0.05), and the scores of MoCA in follow-up were significantly different from those after intervention (*p* < 0.05). The scores of MoCA in sham group after intervention and follow-up were significantly different from those before intervention, and the scores of DSF and DSB after intervention were significantly different from those before intervention (*p* < 0.05). There was no interaction between VFT group and time (F_time*group_ = 2.0410, *p* = 0.149). Further analysis of the main effect showed that the VFT (F_time_ = 20.486, *P* < 0.001) score of patients showed an upward trend before intervention, 4 weeks of intervention and follow-up, but there was no significant difference between the two groups (F_group_ = 0.254, *p* = 0.618) (Table 2).

**Table 2:** Repeated measurement analysis of variance results of cognitive function between the two groups.

Time	MoCA				VFT				DSF				DSB			
	rTMS (*n* = 16)	Sham (*n* = 15)	F	*P*	rTMS (*n* = 16)	Sham (*n* = 15)	F	*P*	rTMS (*n* = 16)	Sham (*n* = 15)	F	*P*	rTMS (*n* = 16)	Sham (*n* = 15)	F	*P*
Pre	11.56 ± 3.63	11.73 ± 3.33	0.019	0.893	6.81 ± 3.12	7.00 ± 2.98	0.029	0.866	4.94 ± 1.57	4.93 ± 1.49	0.000	0.994	2.88 ± 0.72	2.87 ± 0.64	0.001	0.973
Post	17.25 ± 4.85aA	13.73 ± 4.40a	4.450	0.044	9.13 ± 2.78a	8.20 ± 3.82a	0.600	0.445	7.38 ± 1.78aA	5.80 ± 1.97a	5.452	0.027	4.13 ± 0.81aA	3.47 ± 0.83a	4.994	0.033
Follow-up	19.50 ± 5.50abA	14.87 ± 6.37a	4.717	0.038	9.56 ± 3.37a	8.53 ± 4.21a	0.569	0.457	7.56 ± 2.97aA	5.67 ± 2.02	4.263	0.048	4.50 ± 1.41aA	3.40 ± 1.50	4.410	0.045
F	35.776	5.370			16.901	5.182			21.048	2.171			15.219	7.996		
*P*	<0.001	0.02			<0.001	0.22			<0.001	0.133			<0.001	0.005		
Group (F, *P*)	2.796, 0.105				0.254, 0.618				3.561, 0.069				4.409,0.045			
Time (F, *P*)	34.891, <0.001				20.486, <0.001				23.824, <0.001				26.018, <0.001			
Time* group (F, *P*)	7.613, 0.002				2.041, 0.149				5.479, 0.01				4.114, 0.027			

rTMS, repetitive transcranial magnetic stimulation; MoCA, Montreal Cognitive Assessment; VFT, verbal fluency test; DSF, digital span forward; DSB, digital Span Backward. a indicates comparisons with pre, *p* < 0.05; b indicates comparisons with post, *p* < 0.05; A indicates comparisons with the Sham group, *p* < 0.05; both two-way comparisons are Bonferroni corrected.

### 3.3 Repeated measurement analysis of variance results of hormone levels between the two groups before and after intervention and at follow-up

Repeated measurement analysis of variance showed that FT3 group had interaction between group and time (F_time*group_ = 3.610, *p* = 0.04). Further independent effect analysis showed that there was no significant difference in FT3 levels between the two groups at different time points (*p* > 0.05), but there was a significant difference in rTMS group after intervention (*p* < 0.001). There was no interaction between the group of other indicators and time. The independent effect analysis showed that the T3 level in rTMS group was significantly higher than that in sham group after intervention and follow-up (*P*_post_ = 0.046; *P*_follow-up_ = 0.034), Further main effect analysis showed that the levels of T3 (F_time_ = 22.546, *p* < 0.001), FT3 (F_time_ = 6.483, *p* = 0.005), TSH(F_time_ = 12.173, *p* < 0.001) in patients at 4 weeks of intervention and follow-up showed an upward trend, and the effect of improving T3 in rTMS group was better than that in sham group (F_group_ = 5.319, *p* = 0.028) (Table 3).

**Table 3:** Repeated measurement analysis of variance results of hormone levels between the two groups.

Time	T3 (nmol/L)				FT3 (pmol/L)				T4 (nmol/L)				FT4 (pmol/L)				TSH (uIU/mL)			
	rTMS (*n* = 16)	Sham (*n* = 15)	F	*P*	rTMS (*n* = 16)	Sham (*n* = 15)	F	*P*	rTMS (*n* = 16)	Sham (*n* = 15)	F	*P*	rTMS (*n* = 16)	Sham (*n* = 15)	F	*P*	rTMS (*n* = 16)	Sham (*n* = 15)	F	*P*
Pre	0.96 ± 0.05	0.93 ± 0.07	2.638	0.115	3.82 ± 0.56	3.79 ± 0.67	0.012	0.914	118.36 ± 20.24	121.67 ± 20.64	0.203	0.656	12.30 ± 2.14	10.94 ± 2.24	2.974	0.095	1.81 ± 1.23	1.84 ± 1.08	0.004	0.95
Post	1.21 ± 0.20aA	1.07 ± 0.17a	4.327	0.046	4.49 ± 0.93a	3.91 ± 0.71	3.831	0.06	110.49 ± 14.82	115.80 ± 21.71	0.64	0.43	11.55 ± 2.66	10.79 ± 2.37	0.708	0.407	2.77 ± 1.36a	2.56 ± 1.40	0.189	0.667
Follow-up	1.37 ± 0.36abA	1.13 ± 0.24a	4.95	0.034	4.27 ± 1.16	3.96 ± 0.68	0.824	0.371	113.80 ± 20.33	122.01 ± 24.97	1.209	0.281	11.21 ± 1.99	9.68 ± 2.40	3.75	0.063	2.97 ± 1.47a	2.49 ± 1.43	0.829	0.37
F	14.505	10.628			5.304	0.728			1.437	1.278			1.354	2.3			8.66	4.056		
*P*	<0.001	<0.001			0.018	0.492			0.254	0.294			0.273	0.119			0.001	0.039		
Group (F, *P*)	5.319, 0.028				1.488, 0.232				0.839, 0.367				3.724, 0.063				0.747, 0.478			
Time (F, *P*)	22.546, <0.001				6.483, 0.005				2.360, 0.104				3.212, 0.050				12.173, <0.001			
Time*group(F, *P*)	2.092, 0.142				3.610, 0.04				0.347, 0.708				0.370, 0.682				0.747, 0.472			

rTMS, repetitive transcranial magnetic stimulation; T3, triiodothyronine; FT3, free triiodothyronine; T4, thyroxin, FT4, free thyroxine; TSH, thyroid stimulating hormone. Note: a indicates comparison with pre, *P* < 0.05; b indicates comparison with post, *P* < 0.05; A indicates comparison with Sham’s group, *P* < 0.05; both two comparisons are Bonferroni corrected.

### 3.4 Repeated measurement analysis of variance results of MBI between the two groups before and after intervention and at follow-up

Repeated measurement analysis of variance showed that there was no interaction between the group of MBI score and time (F_time*group_ = 2.326, *p* = 0.116). Independent effect analysis showed that there was no significant difference in MBI levels between the two groups at different time points (*p* > 0.05), but the intra-group comparison showed that MBI scores in both groups improved after intervention and at follow-up compared with those before intervention (*p* < 0.05). Further analysis of the main effect showed that the MBI (F_time_ = 23.290, *p* < 0.001) levels of patients before intervention, 4 weeks of intervention and follow-up showed an upward trend, but there was no significant difference between the two groups (F_group_ = 1.999, *p* = 0.168) (Table 4).

**Table 4:** Repeated measurement analysis of variance results of MBI between the two groups.

Time	rTMS (*n* = 16)	Sham (*n* = 15)	F	*P*
Pre	46.94 ± 8.18	44.67 ± 9.29	0.524	0.475
Post	57.75 ± 10.48^a^	50.27 ± 13.57^a^	2.975	0.095
Follow-up	61.06 ± 16.34^a^	52.13 ± 18.22^a^	2.069	0.161
F	20.666	6.585		
*P*	<0.001	0.010		
Group (F, *P*)	1.999, 0.168			
Time (F, *P*)	23.290, <0.001			
Time*group(F, *P*)	2.326, 0.116			

Unit: points. rTMS, repetitive transcranial magnetic stimulation; MBI, Modified Barthel index. a indicates comparisons with pre, *P* < 0.05; both two-way comparisons are Bonferroni corrected.

### 3.5 Comparative analysis of cognitive change values (after 4 weeks of intervention-pre-intervention) between the two groups

Mann Whitney U test was used to compare the changes of cognitive function (after 4 weeks of intervention pre intervention) between the two groups. The results showed that after 4 weeks of intervention, the differences of cognitive function and MBI scores in rTMS group were higher than those in sham group, with statistical differences (*p* < 0.05) (Table 5).

**Table 5:** Comparative analysis of the difference of cognitive change values (after 4 weeks of intervention-pre-intervention) between the two groups.

Group	MoCA	VFT	DSF	DSB	MBI
rTMS (*n* = 16)	6 (1, 10)A	2 (−1, 5)A	2 (1, 5)A	1 (0, 3)A	9.50 (2, 25)A
Sham (*n* = 15)	2 (−5, 6)	1 (−2, 4)	1 (−1, 3)	1 (-1, 1)	5.50 (-6, 21)
Z	−3.056	−2.328	−2.903	−2.139	−2.002
*P*	0.002	0.020	0.004	0.032	0.045

Unit: points. rTMS, repetitive transcranial magnetic stimulation; MoCA, Montreal Cognitive Assessment; VFT, verbal fluency test; DSF, digital span forward; DSB, digital Span Backward. A indicates comparison with the Sham group, *p* < 0.05.

### 3.6 Comparative analysis of hormonal change values (after 4 weeks of intervention-pre-intervention) between the two groups

Mann Whitney U test was used to compare the changes of thyroid hormone levels (after 4 weeks of intervention pre intervention) between the two groups. The results showed that after 4 weeks of intervention, the difference of FT3 levels in rTMS group was higher than that in sham group, with statistical difference (*p* = 0.024) (Table 6).

**Table 6:** Comparative analysis of the difference of hormonal change values (after 4 weeks of intervention-pre-intervention) between the two groups.

Group	T3 (nmol/L)	FT3 (pmol/L)	T4 (nmol/L)	FT4 (pmol/L)	TSH (uIU/mL)
rTMS (*n* = 16)	0.21 (−0.04, 0.54)	0.53 (−0.28, 2.54)A	−8.85 (−51, 22.4)	−0.78 (−6.45, 3.97)	1.11 (−2.04, 2.86)
Sham (*n* = 15)	0.13 (−0.06, 0.43)	0.11 (−0.67, 1.00)	−9.66 (−20.21, 38.29)	−0.55 (−4.23, 3.82)	0.82 (−2.04, 2.86)
Z	−1.682	−2.254	−0.514	−0.435	−0.909
*P*	0.093	0.024	0.607	0.664	0.363

rTMS, repetitive transcranial magnetic stimulation; T3, triiodothyronine; FT3, free triiodothyronine; T4, thyroxin, FT4, free thyroxine; TSH, thyroid stimulating hormone. A indicates comparison with the Sham group, *p* < 0.05.

### 3.7 Correlation between changes in thyroid hormone levels and cognitive function scores in rTMS group patients

**Figure 2:**
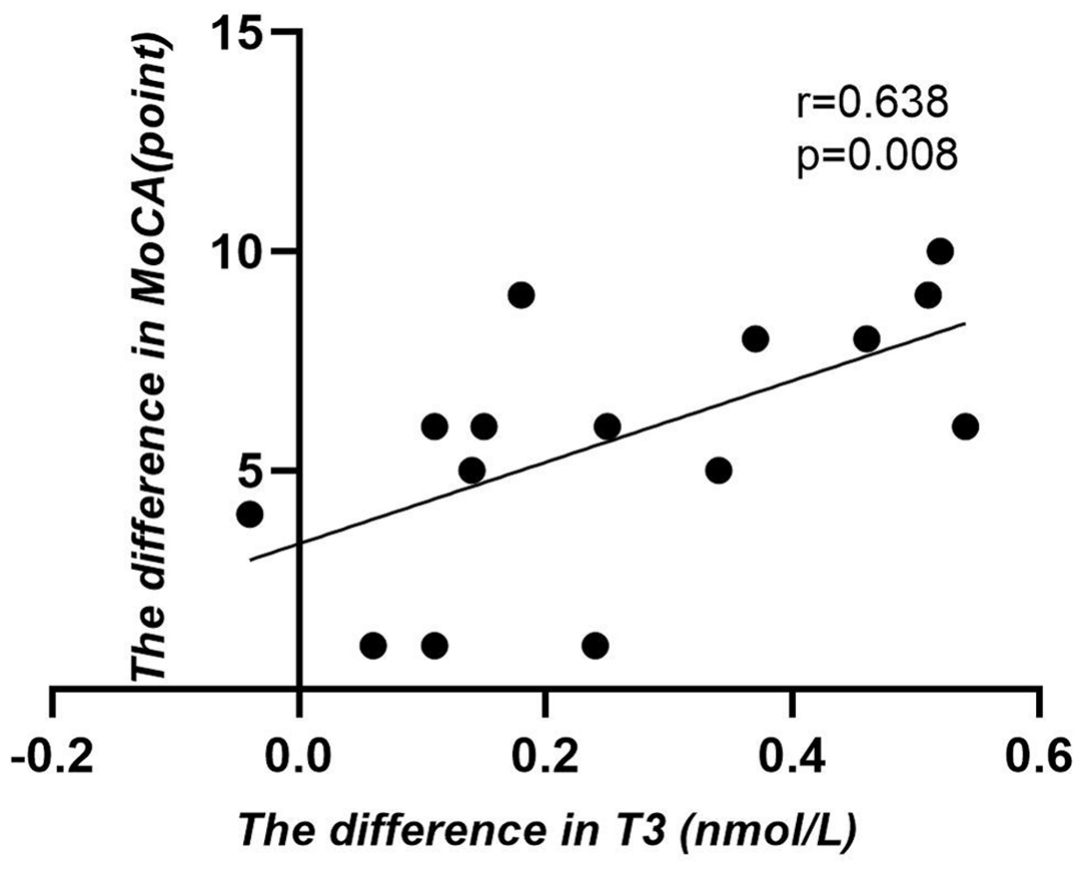
Scatter plot analysis of the correlation between T3 levels and MoCA scale (post-pre) in rTMS group.

(1) Correlation between the value of changes in various indicators of thyroid hormone levels and the value of changes in the scores of various indicators of cognitive function (after 4 weeks of intervention - before intervention) in patients in the rTMS group: the results of the analysis using Spearman correlation analysis showed that the value of changes in the levels of T3 in the rTMS group of patients and the value of changes in the scores of MoCA were in a positive correlation relationship after 4 weeks of intervention (*r* = 0.638, *p <* 0.05) (Table 7, Figure 2).
(2) Correlation between changes in thyroid hormone levels and changes in MoCA cognitive domain scores (after 4 weeks of intervention - before intervention) in the rTMS group: Spearman’s correlation analysis showed that there was a positive correlation between changes in T3 levels and changes in naming, delayed recall, and attention scores in the rTMS group after 4 weeks of intervention (*r* = 0.562, *p <* 0.05; *r* = 0.562, *p <* 0.05; *r* = 0.531, *p <* 0.05); and the value of change in FT3 level showed a positive correlation with the value of change in visuospatial and executive function scores (*r* = 0.514, *p* < 0.05) (Table 8).

**Table 7:** Correlation analysis between values of hormonal changes and values of changes in cognitive scores (after 4 weeks of intervention-pre-intervention) in the rTMS group(r, *P*).

	MoCA	VFT	DSF	DSB	MBI
T3 (nmol/l)	0.638, 0.008^**^	0.018, 0.946	0.434, 0.093	0.242, 0.367	0.028, 0.918
T4 (nmol/l)	−0.288, 0.279	0.217, 0.419	−0.058, 0.831	−0.066, 0.809	0.396, 0.128
FT3 (pmol/l)	0.119, 0.662	0.338, 0.201	−0.031, 0.911	0.175, 0.516	0.189, 0.484
FT4 (pmol/l)	−0.014, 0.960	−0.135, 0.617	0.168, 0.534	0.128, 0.636	−0.228, 0.395
TSH (uIU/mL)	0.215, 0.425	−0.050, 0.854	0.211, 0.434	0.050, 0.854	−0.352, 0.181

^**^P<0.01. Abbreviations: rTMS, repetitive transcranial magnetic stimulation; MoCA, Montreal Cognitive Assessment; VFT, verbal fluency test; DSF, digital span forward; DSB, digital Span Backward; MBI, Modified Barthel index; T3, triiodothyronine; FT3, free triiodothyronine; T4, thyroxin, FT4, free thyroxine; TSH, thyroid stimulating hormone.

**Table 8:** Correlation analysis of hormonal indicators in the TMS group with the difference in change in MoCA scores in each cognitive domain (after 4 weeks of intervention—before intervention) (r, *P*).

	Visuo-spatial and executive functions	Naming	Delayed recall	Attention	Language	Abstraction thinking	Orientation
T3 (nmol/l)	0.277, 0.300	0.562, 0.024*	0.562, 0.024*	0.531, 0.034*	0.260, 0.330	0.085, 0.756	0.327, 0.216
T4 (nmol/l)	0.203, 0.452	−0.252, 0.346	−0.370, 0.158	−0.455, 0.077	0.096, 0.725	−0.270, 0.312	0.067, 0.805
FT3 (pmol/l)	0.514, 0.042*	0.308, 0.246	0.106, 0.697	0.109, 0.686	0.232, 0.387	0.428, 0.098	0.301, 0.257
FT4 (pmol/l)	−0.393, 0.133	0.196, 0.467	−0.187, 0.489	0.014, 0.960	0.205,0.446	0.162, 0.549	−0.276, 0.301
TSH (uIU/ml)	0.185, 0.492	0.028, 0.918	0.096, 0.724	0.211, 0.432	0.178, 0.510	0.333, 0.208	0.042, 0.878

^*^P<0.05. rTMS, repetitive transcranial magnetic stimulation; T3, triiodothyronine; FT3, free triiodothyronine; T4, thyroxin, FT4, free thyroxine; TSH, thyroid stimulating hormone.

## 4 Discussion

There are many studies relating to thyroid hormone levels to cognitive function after stroke (Gkantzios et al., 2023; Lei et al., 2019; Li et al., 2021; Murolo et al., 2022), and it has been concluded that the majority of patients with post-stroke cognitive dysfunction have abnormal thyroid hormone levels, and that persistent low thyroid hormone levels exacerbate the dysfunction of the PSCI. However, very few studies have examined clinical interventions within this specific population. Our study aims to find a safe and effective rehabilitation method for this population to reduce the adverse effects of chronic low thyroid hormone in older patients with cognitive impairment after stroke. A large number of studies have shown that low-frequency repetitive transcranial magnetic stimulation is effective in improving cognitive dysfunction in post-stroke patients through central mechanisms (Blesneag et al., 2015; Gong et al., 2023; Wang et al., 2024; Yingli et al., 2022), and some animal studies have also found that rTMS improves endocrine hormones and cognitive function in naturally aging mice from the perspectives of synaptic plasticity and neurophysiology, suggesting that rTMS improves cognitive function through a potential neuroendocrine mechanism (Ren et al., 2017; Zhu et al., 2020). As research advances in the field of neuroendocrinology, clinical studies have demonstrated that rTMS can increase cerebral blood flow in healthy elderly people and stroke patients, in addition to increasing T3 levels in the peripheral blood, suggesting that rTMS may regulate the disorders of the hypothalamic–pituitary-thyroid axis and elevate the hormone levels to improve cognition through a potential neuroendocrine mechanisms (Ma et al., 2021; Mei et al., 2022). The results of the present study showed that rTMS significantly improved both cognition and T3 levels in PSCI patients with low thyroid hormone levels, furthermore, compared with the Sham group, the improvement effects of rTMS on cognitive function and T3 levels were statistically different at 4-week intervention as well as at follow-up, and T3, MoCA, DSF, and DSB continued to improve with rTMS at follow-up, compared with pre-intervention as well as post-intervention, suggesting that there is a sustained effect of rTMS. Based on current research advances on the central and neuroendocrine mechanisms by which rTMS improves cognitive function. We hypothesise the following possible reasons for the improvement of thyroid hormone levels and cognitive function with rTMS: on the one hand, the DLPFC, as a target area threshold that closely related to both cognitive function (Fischer et al., 2013) and is also a major area of thyroid hormone action. Stimulation of this area may affect both cognition and thyroid hormone levels; on the other hand, based on the central mechanism by which rTMS improves cognition, stimulation of the DLPFC increases cerebral blood flow and metabolic levels, promotes the release of neurotransmitters, and increases signaling, thereby enhancing the neuronal connections between the cortex and hypothalamus (Basilis et al., 2007) thus affecting the HPT axis and causing changes in THs levels. In addition, rTMS promotes the recovery of cognitive function, which will directly affect the level of other bodily functions of the organism, and overall improves the metabolic function level of the organism, which in turn affects hormone levels.

Based on the results of repeated measures ANOVA, T3 levels as well as cognitive levels were statistically different in the rTMS group compared to the Sham group, both at post-intervention and at follow-up (*P* < 0.05), our study also investigated whether changes in cognitive function scores observed after rTMS treatment were correlated with equivalent changes in thyroid hormone levels in older stroke patients with low thyroid hormone levels. These investigations showed that among patients who received rTMS treatment, increases in T3 levels were significantly and positively correlated with increases in MoCA scores that were observed after 4 weeks of rTMS treatment was delivered (*r* = 0.638, *p* < 0.05). It is appears that within a certain range, the change of T3 level in early older stroke recovery patients with cognitive dysfunction and low thyroid hormone level is correlated with the change in the MoCA score, i.e., the cognitive function of patients will be improved together with the increase of T3 level, and the increase of T3 level will, in turn, improve the cognitive function of patients with cognitive dysfunction in stroke, and that the two function will boost each other and influence each other. In addition, specific scores for naming, delayed recall, and attention functions of patients in the rTMS group improved with increasing T3 levels, and visuospatial and executive function scores improve with increasing FT3 levels. Improvements in these cognitive domain scores may have contributed to the increase in overall MoCA values. We hypothesise that there are multiple factors that are likely to have contributed to this observation: (1) In peripheral blood, 80–90% of T3 is converted from T4 by deiodinase, and the clinical stress of stroke inhibits this deiodinase conversion and thereby reduces levels of T3, which is the main thyroid hormone that exerts a wide range of biological effects, including effects on the nervous system (Bunevicius et al., 2016; Chen et al., 2016; Lamba et al., 2018; Wang et al., 2017). Reduced T3 may further exacerbate cognitive deficits and reduce an individual’s abilities to be independent in daily life. (2) The cerebral cortex and hippocampus are brain regions that are closely related to cognitive function and are also the main sites of thyroid hormone action. When cerebrovascular damage to specific neural networks or circuits occurs, cognitive function will be directly affected (Chen et al., 2016; Mei et al., 2022) and, at the same time, the biological effects of thyroid hormones will be inhibited, and abnormal levels of thyroid hormones may exacerbate cognitive dysfunction. (3) It has been found that the blood–brain barrier is disrupted after stroke, which affects the level of organic anion-transporting polypeptides in epithelial cells, thus causing a decrease in the levels of peripheral thyroid hormones that are responsible for their transport, and a decrease in T3 levels in the brain. This decrease in T3 levels will affect cerebral tissue blood perfusion, energy metabolism (Zhu et al., 2020) and signal systems, in addition to interfering with the generation of synaptic long-range potentiation (Hainsworth et al., 2017; Van Heugten et al., 2017), which in turn affects cognitive function.

This study has certain limitations, such as a small sample size, incomplete evaluation methods, and a lack of in-depth exploration of the specific mechanisms by which rTMS improves cognition and thyroid hormone levels in older early stroke patients with low thyroid hormone levels. In the future, large-scale multicenter randomized controlled trials should be conducted, and more objective evaluation methods such as brain imaging technology should be used to explore the potential neuroendocrine mechanisms, and long-term tracking of the functional prognosis of this group of people is needed, which will provide new ideas for early clinical intervention in patients with low thyroid hormone levels in PSCI, and minimize the adverse effects of long-term low thyroid hormone levels.

## 5 Conclusion

rTMS can effectively improve the cognitive function of early stroke patients with low thyroid hormone levels in addition to elevating their low thyroid hormone levels, and the therapeutic has a sustained effect, which is superior to a single conventional cognitive training. Within the normal range, changes in T3 levels are positively correlated with changes in cognitive functioning. Moreover, there were no adverse reactions and other conditions during the whole study, suggesting that this approach is safe for clinical application. Clinical attention should be paid to older stroke patients with low thyroid hormone levels as early as possible, and timely monitoring and targeted interventions should be provided to reduce the risk that low thyroid hormone may further aggravate the degree of cognitive impairment in stroke patients.

## Data Availability

The datasets presented in this study can be found in online repositories. The names of the repository/repositories and accession number(s) can be found in the article/supplementary material.
